# Phenotypic and biochemical responses of high-yield tomato varieties to salinity and drought stress

**DOI:** 10.1371/journal.pone.0355714

**Published:** 2026-08-10

**Authors:** Shahida Ferdousee, Myung Hwangbo, Md Sakil Arman, Muhammad Abul Kalam Azad, Ajit Ghosh, Jongsun Kim

**Affiliations:** 1 School of Earth, Environmental and Marine Sciences, The University of Texas Rio Grande Valley, Brownsville, Texas, United States of America; 2 Department of Biochemistry and Molecular Biology, Shahjalal University of Science and Technology, Sylhet, Bangladesh; 3 School of Earth, Environmental and Marine Sciences, The University of Texas Rio Grande Valley, Edinburg, Texas, United States of America; 4 Department of Agriculture, University of Arkansas at Pine Bluff (UAPB), Arkansas, United States of America; South Asian University, INDIA

## Abstract

Abiotic stresses can influence plant growth and productivity by causing physiological, biochemical, molecular, and morphological changes. Salinity and drought are increasing in frequency and intensity because of climate change. Therefore, this study assessed four high-yield tomato cultivars under 150 mM NaCl and 260 mM mannitol treatments to characterize physiological (i.e., chlorophyll level, root/shoot length, and relative water content) and biochemical responses (i.e., GLY I/II and DLDH enzyme activities, proline, and hydrogen peroxide). Results indicated that the four BARI tomato varieties showed genotype- and treatment-specific physiological and biochemical responses rather than a single uniform tolerance pattern. BARI tomato 2 maintained relatively higher shoot growth and showed strong proline accumulation under stress, whereas BARI tomato 16 showed higher chlorophyll retention, RWC, and lower H_2_O_2_ accumulation. GLY I, GLY II, and DLDH activities were significantly affected by variety, treatment, and their interaction, with reduced activities in several stress treatments after 120 h exposure. Moreover, there was a significant accumulation of proline and hydrogen peroxide in the plant leaves. These results showed that MG detoxification-related enzyme activities are useful biochemical markers for comparing tomato responses to salinity and drought. Thus, this research can provide stress-response profiles of high-yielding BARI tomato varieties at the seedling stage and reveal DLDH as an unexplored downstream component of MG detoxification-related metabolism during salinity and drought stress.

## 1. Introduction

Plants go through a lot of diverse environmental interactions during their growth which can be classified into biotic and abiotic factors [1]. Abiotic stresses such as salinity, drought, and fluctuating temperatures are some of the leading causes of global crop losses [2]. Salinity and drought are becoming more frequent and severe due to climate change, especially in arid and semi-arid regions consisting of nearly half of the world [3,4]. By 2050, about half of the world’s cultivable land is expected to be salinized in these regions [5]. Evaporation in coastal and dry regions increases soil salinity, linking drought and salt stress [6]. Accordingly, understanding the phenotypic stress responses of plants is necessary for improving agricultural productivity and ensuring food security in regions affected by drought and salinity [3].

These drought and salt stresses can affect plant growth and productivity by altering the physiological, biochemical, molecular as well as morphological alterations [7]. Although researchers cannot pinpoint the primary regulatory component involved because of the complexity of metabolic pathways and their regulation, plants often produce harmful aldehydes in the stress response [8]. Activities of enzymes (i.e., GLY I/II and DLDH), accumulation of osmolytes (i.e., proline), and oxidative stress markers (i.e., hydrogen peroxide (H_2_O_2_)) are phenotypic parameters that span growth and production of plant stress responses. Methylglyoxal (MG), a cytotoxic by-product of glycolysis routes, forms advanced glycation end-products that damage proteins and nucleic acids under salinity and drought conditions [9,10]. Plants detoxify MG through a glutathione (GSH) dependent glyoxalase system [11]. The sequential activity of glyoxalase I (GLY I) and glyoxalase II (GLY II) enzymes reacts with MG and produces D-lactate as the ultimate product with the activity of reduced GSH [12]. GLY I converts a nonenzymatically formed complex of MG and GSH into S-d-lactoylglutathione (SLG). Then, GLY II breaks down SLG, releasing GSH and producing D-lactate. Then, D-lactate dehydrogenase (DLDH) is another enzyme that can convert D-lactate to pyruvate and then go to the tricarboxylic acid (TCA) cycle [13–15].

Proline, as another stress marker, accumulates as both an osmoprotectant and reactive oxygen species (ROS) scavenger, stabilizing proteins and membranes without cytotoxic effects [16–19]. Also, although the production of reactive oxygen species (ROS) (i.e., singlet oxygen (^1^O_2_), superoxide (O_2_^●−^), hydroxyl radicals (OH^●^), and hydrogen peroxide (H_2_O_2_)) are necessary for plant physiology and fundamental biological processes, research showed that these molecules can also serve as stress markers [20,21]. Accordingly, integrating measurements of these stress-marker activities enables a comprehensive understanding of how specific biochemical and oxidative responses influence plant growth and productivity under abiotic stress.

Tomatoes are one of the most favored vegetables grown worldwide, with rising consumption and production due to the antioxidant and anti-cancer benefits of lycopene [22]. However, its productivity is affected by various abiotic stresses, making the development of stress-resistant crops a major goal in agricultural biotechnology [23]. In addition to its agricultural value, tomato is also used as a model plant for studying stress tolerance because of its physiological characteristics and its relationship with other Solanaceae crops such as potato, eggplant and peppers, making it possible to transfer the knowledge gained from tomato research to other crops in the family. Recent advancements in tomato genome sequencing have yielded extensive genetic and molecular data that enhance our knowledge of stress responses across genomic and physiological dimensions [24]. However, comparative phenotypic profiling across high-yield tomato cultivars remains lacking while salinity and drought significantly limit tomato yield. By learning how tomatoes respond to drought and salinity, it will be easier to develop resilient crop varieties along with sustainable farming practices that can endure these challenging conditions.

This study provides a comparative phenotypic and biochemical assessment of four high-yield tomato varieties under controlled salinity and drought stress. The physical effects such as root length, shoot length, with relative water content and chlorophyll, as well as the MG detoxifying enzymes such as GLY I, GLY II, DLDH, and some other stress indicators, including proline and hydrogen peroxide, were measured in this study. The phenotypic profiling of this study is expected to fill the gap of limited data on DLDH activity under stress in tomatoes and inform future mechanistic research. Thus, this study will help better understand plant physiological responses and contribute to the development of stress-tolerant tomato varieties.

## 2. Materials and methods

### 2.1. *Chemicals*

Sodium chloride (Himedia, India), mannitol (Himedia, India), Potassium dihydrogen phosphate (Himedia, India), Potassium phosphate dibasic (Himedia, India), glycerol (Himedia, India), magnesium sulfate (Himedia, India), phenylmethylsulfonyl fluoride (PMSF) (Himedia, India), Methylglyoxal (MG) (Himedia, India), reduced glutathione (GSH) (Himedia, India), S-D-Lactoylglutathione (SLG) (Himedia, India), MOPS (Himedia, India), phenazine methosulfate (PMS) (Himedia, India), 2,6 dichlorophenolindophenol (DCIP) (Himedia, India), sodium D-lactate (Sigma Aldrich, St. Louis, MA), sulphosalicylic acid (Himedia, India), acetic acid (Himedia, India), ninhydrin (Himedia, India), Dimethyl formamide (DMF) (Himedia, India), trichloroacetic acid (TCA) (Himedia, India), potassium iodide (KI) (Himedia, India).

### 2.2. *Stress treatment*

Four high-yielding winter tomato varieties were collected from the Bangladesh Agricultural Research Institute (BARI), Bangladesh, namely BARI tomatoes 2, 15, 16, and 18 (https://bari.gov.bd/). All seedlings were grown under the same conditions with a 14-h light/10-h dark cycle at a temperature of 26 ± 2 °C. Stress treatments were applied to 7-day-old seedlings using NaCl to simulate 150 mM salinity and 260 mM mannitol to simulate drought, as outlined in Table 1, to induce significant stress levels without causing immediate cell death [25]. Samples were collected after 5 days to assess the effect of long-term stress [26] on enzyme activity in comparison to untreated samples.

**Table 1:** Chemicals used to mimic stress treatments.

Treatment	150 mM NaCl	260 mM Mannitol
Control	No	No
Salinity	Yes	No
Drought	No	Yes

### 2.3. *Measurement of plant growth and water content*

The lengths of plants’ shoots and roots were measured 12 days after sowing. The weights of the shoots and roots were determined through the process of placing the plants at 100°C (i.e., a hot air oven) for a duration of 2 hours. Relative Water Content (RWC) was assessed following the methods of Barrs and Weatherley [27]. Cut leaves were weighed to obtain Fresh Weight (FW), then left to saturate in distilled water in a closed petri dish for three hours to determine their Turgid Weight (TW). After this period, the leaf samples were subjected to a preheated oven set at 80°C for a duration of 24 hours to get the Dry Weight (DW). The values of FW, TW, and DW were used to calculate RWC, using the following equation:


$$\begin{array}{c} RWC=\;\frac{FW-DW}{TW-DW}\times \text{1}\text{0}\text{0} \end{array}$$

### 2.4. *Total protein extraction*

The total protein was extracted from fresh tomato tissue (~200 mg) making it powdered and homogenized with ice-cold extraction buffer containing 100 mM potassium phosphate buffer with pH 7, 50% glycerol, 16 mM MgSO_4,_ and 0.5 mM PMSF. The homogenate underwent centrifugation at a rate of 12,000g for 30 min at 4°C [28]. The protein was quantified using Bradford method [29].

### 2.5. *Assay of methylglyoxal detoxifying enzymes*

GLY I activity was measured conventionally in the spectrophotometer, using a solution containing 0.1M sodium phosphate buffer with pH 7.5, 3.5 mM MG, 1.7 mM reduced glutathione (GSH), and 16 mM MgSO_4_. The absorbance was measured at 240 nm as GLY I enzyme catalyzes the reaction where 1 μmol of SLG is produced per minute from GSH [30]. The hydrolysis of SLG from a reaction mixture of 10 mM MOPS, 300 μM SLG, and 500 ng protein was used to measure GLY II activity. The absorbance was observed at 240 nm [30]. DLDH activity was determined via spectrophotometric analysis utilizing a reaction mixture comprised of 50 mM potassium phosphate buffer within a pH range of 6.0–9.5 and 3 mM phenazine methosulfate (PMS) and 200 μM 2,6 dichlorophenolindophenol (DCIP) and 10 mM of sodium D-lactate as substrates. Activities were measured at 600 nm [31].

### 2.6. *Measurement of proline, chlorophyll, and H*_*2*_*O*_*2*_ *Level*

To estimate the proline content, a crude extract was obtained from 200 mg of fresh leaf tissue. The tissue was ground and homogenized with 100 mM phosphate buffer at pH 7.8. The mixture was subsequently subjected to centrifugation, and the supernatant was collected for proline measurement. The reaction mixture was composed of 3% sulphosalicylic acid, acetic acid, and 2.5% ninhydrin solution, which was mixed with the crude extract. After boiling and cooling, the absorbance was read at 520 nm. The proline content was determined by utilizing a standard curve consisting of proline concentrations ranging from 0 to 100 nmoles [13]. For chlorophyll estimation, 100 mg of fresh leaf tissue was ground into powder through the use of liquid nitrogen and then homogenized in 1 mL of Dimethyl formamide (DMF). Then the extract was centrifuged, and supernatant optical density was measured at 645 nm, 663 nm, and 470 nm [32,33].

The H_2_O_2_ contents were measured spectrophotometrically after preparing the leaf extract supernatant, homogenizing with 1mL of 0.1% trichloroacetic acid (TCA). After that, the reaction mixture was prepared with 200 μL of the leaf extract supernatant, 300 μL of 100 mM potassium phosphate buffer adjusted to pH 7.8, and 1 mL of reagent (1 M KI, *w*/*v* in freshly prepared double-distilled water). For the blank control, the reaction mixture was prepared using the same reagents except the leaf supernatant and instead 500 μL of 1 M potassium iodide (KI) was used. Then, all the reaction mixtures were incubated in the dark for 1 hour, after which the absorbance was recorded at a wavelength of 390 nm. The concentration of H_2_O_2_ was measured utilizing a standard curve prepared with an established level of H_2_O_2_ [34].

### 2.7. *Statistical analysis*

Replicate-level data were used for all statistical analyses. The number of replicate measurements ranged from n = 3 to n = 4 depending on the parameter and variety-treatment combination. Statistical analyses were performed using RStudio. Tomato variety, stress treatment, and variety × treatment interaction effects for each parameter were determined by two-way analysis of variance (ANOVA). Pairwise comparisons between variety-treatment combinations were performed using Tukey’s honestly significant difference (HSD) test. For graphical presentation, bars show mean values, and error bars indicate standard deviation. Different lowercase letters in the figures indicate statistically significant differences. For GLY I activity, the two blank-derived zero values were treated as missing data and excluded from the analysis. Residual normality and homogeneity-of-variance tests were used to assess model assumptions. For parameters that did not fully meet ANOVA assumptions, nonparametric and robust sensitivity analyses were performed to confirm whether the main conclusions were consistent. Statistical significance was set at p < 0.05.

### 2.8. *Inclusivity in global research*

Additional information regarding the ethical, cultural, and scientific considerations specific to inclusivity in global research is included in the Supporting Information (S1 Checklist).

## 3. Results and discussion

### 3.1. *Effect of salinity and drought on plant growth, relative water content, and Chlorophyll level*

**Fig 1:**
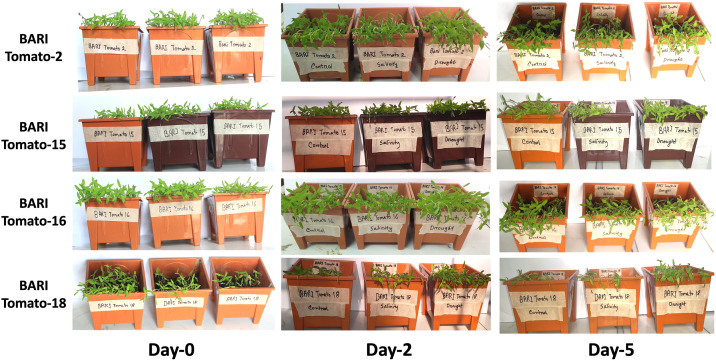
Visual comparison of high-yield tomato varieties BARI tomatoes 2, 15, 16, and 18 under salinity, drought stress treatments and control without any stress treatments. Pictures were taken from 7-day-old seedlings before stress treatment and up to day 5 after stress treatment.

**Fig 2:**
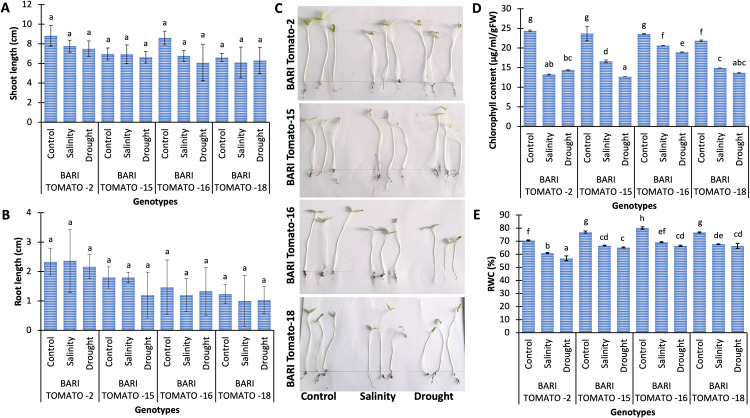
Effect of stress treatments on physiological parameters under salinity, drought, and control without any stress treatments on tomato varieties: (A) Shoot length (B) Root length (C) Visual representation of plants (D) Chlorophyll content, and (E) Relative Water Content (RWC) %. Different lowercase letters stand for significant differences among variety-treatment combinations based on Tukey’s HSD test (p < 0.05). For shoot length, root length, total chlorophyll content, and RWC, n = 3 per variety-treatment combination. Bars and error bars stand for mean values and standard deviations, respectively.

**Fig 3:**
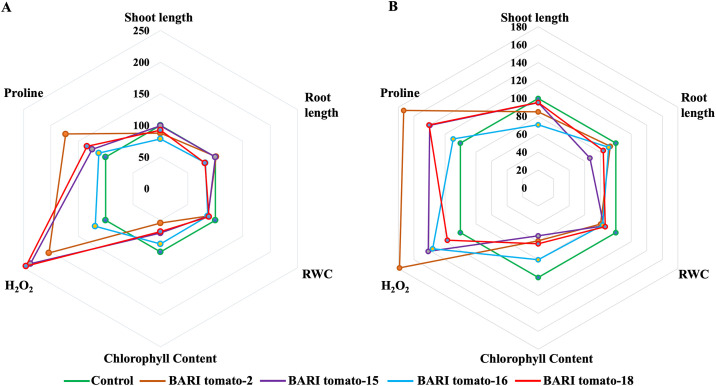
Impact of stress treatment on plant root, shoot length, relative water content, H_2_O_2_, and proline content (A) comparison between salinity with control (no stress treatment) (B) comparison between drought with control (no stress treatment) on tomato varieties. Values are presented as percentage responses relative to the corresponding control condition.

Plants’ response to abiotic stress conditions is studied widely in various species; however, as the root is directly exposed to the stress condition, the response of plants toward the stress conditions varies from species to species, along with the kind of stress (Fig 1) [35]. In this study, four high-yielding tomato varieties, BARI tomatoes 2, 15, 16, and 18, were compared under control, salinity, and drought conditions. Moreover, the first physical appearance of stress is observed by root and shoot growth [36]. Our study showed that both salinity and drought conditions affected the shoot length, with significant effects of tomato variety and stress treatment (p < 0.05), whereas the variety × treatment interaction was not significant (Fig 2A). Although the main effects of variety and treatment were significant, Tukey’s HSD comparisons did not separate the variety-treatment combinations into different statistical groups for shoot length. Additionally, Fig 3 shows the four tomato varieties’ relative responses to salinity (Fig 3A) and drought stress (Fig 3B) compared to control plants. Shoot length was generally reduced by salinity and drought stress. BARI tomato 2 had the greatest mean shoot length over all treatments, with values of 8.83 cm, 7.77 cm, and 7.50 cm for control, salinity, and drought treatments, respectively. BARI tomato 16 had the greatest relative decrease in shoot length due to drought stress, with about a 29% decrease from the control.

Root length did not vary as much between varieties, and two-way ANOVA showed a significant variety effect, but treatment and variety × treatment interaction effects were not significant (Fig 2B). Tukey’s HSD comparisons also did not show significant separation among variety-treatment combinations for root length. Although root length decreased in some varieties under stress, including an approximately 17% decrease in BARI tomato 2 under drought, these treatment-related differences were not statistically significant. Overall, both stress conditions negatively impacted root and shoot lengths, but the statistical response was clearer for shoot length than for root length. A previous study showed that drought stress usually affects shoot growth more than root growth [37] due to their rapid osmotic adjustment to decreased soil water content, maintaining water uptake, and the increased loosening ability of root cell walls [38]. Moreover, during the salinity and drought conditions, root length was decreased compared to control conditions in several varieties (Figs 2B and 3), but these treatment-related differences were not statistically significant (p > 0.05). This indicates that shoot length was more responsive to stress treatment than root length in the tested tomato varieties.

Abiotic stress also affects the chlorophyll content of the exposed plants. In this study, both salinity and drought conditions significantly decreased the amount of total chlorophyll in exposed plants (Figs 2D and 3). Total chlorophyll content was significantly affected by tomato variety, stress treatment, and the variety × treatment interaction (p < 0.05). Although the direction of chlorophyll response was generally negative under stress, the magnitude of reduction differed among varieties and treatments. BARI tomato 16 showed higher amounts of total chlorophyll under abiotic stress compared to other varieties, particularly in salinity condition (Fig 3A). BARI tomato 16 retained 20.62 µg/mL/g FW under salinity and 18.93 µg/mL/g FW under drought, corresponding to reductions of approximately 13% and 20%, respectively, compared with the control. In contrast, BARI tomato 2 showed a stronger chlorophyll reduction under salinity, decreasing by approximately 46%, while BARI tomato 15 showed the strongest reduction under drought, decreasing by approximately 47%. Moreover, a decrease in chlorophyll can result from decreased water content, resulting in ROS production [39]. As chlorophyll plays an important role in photosynthesis and plant biomass production, a decrease in chlorophyll may result in reduced growth, biomass, as well as other metabolic activities [40].

Measuring RWC is the easiest method to measure plant drought as well as salinity stress [39]. This study also showed that RWC was decreased in both salinity and drought conditions (Fig 3), with significant effects of tomato variety, stress treatment, and the variety × treatment interaction (p < 0.05). BARI tomato 15, 16, and 18 varieties showed comparatively higher RWC than BARI tomato 2 (Fig 2E). However, BARI Tomato-16 under drought stress exhibited relatively higher RWC (Fig 3B). BARI tomato 16 had the highest RWC under control conditions (80.16%), while BARI tomato 2 had the lowest RWC under both salinity and drought, with 61.01% and 56.97%, respectively. These results suggest that water-retention capacity differed among varieties and that BARI tomato 2 maintained relatively higher shoot and root growth despite lower RWC under stress. The lower RWC can induce osmotic stress in plants [41]. Thus, the growth, chlorophyll, and RWC results show that the varieties differed in stress response traits. BARI tomato 2 showed relatively favorable growth responses, whereas BARI tomato 16 showed stronger chlorophyll retention and water-status responses under stress.

### 3.2. *Effect of salinity and drought on methylglyoxal detoxifying enzymes*

**Fig 4:**
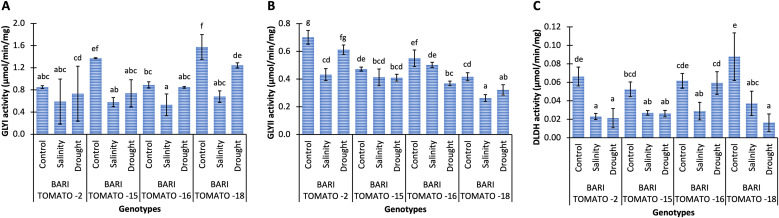
Effect of stress treatments on methylglyoxal detoxifying enzyme activity under salinity, drought, and control without any stress treatments on tomato varieties: (A) Glyoxalase I (GLY I) (B) Glyoxalase II (GLY II) (C) D-lactate dehydrogenase (DLDH). Different lowercase letters indicate significant differences among variety-treatment combinations based on Tukey’s HSD test (p < 0.05). For GLY I, n = 3-4 depending on the variety-treatment combination; for GLY II and DLDH, n = 4 per variety-treatment combination. Bars and error bars stand for mean values and standard deviations, respectively.

MG acts as an important signaling molecule for plants. However, under different abiotic stresses such as salinity and drought, the MG levels can increase up to 2–6 folds [42]. If this excess MG accumulates in plant body, it is harmful as well [9]. To understand the response of the MG detoxification-related pathway under abiotic stress, we measured GLY I, GLY II, and DLDH activity along with control samples (Fig 4). In our study, the result showed that in the salinity and drought conditions, MG detoxification-related enzyme activities generally decreased compared to the control, but the magnitude of the response differed among tomato varieties and stress treatments. Two-way ANOVA revealed that tomato variety, stress treatment and the interaction between them had significant effects on GLY I, GLY II and DLDH activities (p < 0.05), suggesting variety-specific biochemical responses to salinity and drought stress.

There was a significant decrease of GLY I in salinity conditions compared to the control samples; especially, GLY I activity was decreased by approximately 57% in BARI tomato 18 and 58% in BARI tomato 15 compared to the control (Fig 4A). BARI tomato 16 also showed an approximately 40% decrease in GLY I activity under salinity. In contrast, BARI tomato 2 showed only a small decrease under salinity and maintained GLY I activity under drought, suggesting that GLY I activity was not uniformly suppressed across all varieties. For GLY II enzyme activity, BARI tomato 2 and BARI tomato 18 showed clear reductions under salinity stress (Fig 4B). Under salinity conditions, GLY II enzyme activity was decreased up to 38% and 37% in BARI tomatoes 2 and 18, respectively, compared to the control. Under drought, GLY II activity decreased by approximately 13% in BARI tomato 2 and 23% in BARI tomato 18, whereas BARI tomato 16 showed a stronger drought-associated reduction of approximately 33%. Interestingly, DLDH enzyme activity showed significant differences for both salinity and drought conditions compared to the control samples. Particularly, DLDH enzyme activity was the most reduced by about 81% in BARI tomato 18 under drought conditions compared to the control (Fig 4C). DLDH activity in BARI tomato 2 was also significantly reduced upon salinity and drought treatments. The average activity was reduced by around 65% and 68% for salinity and drought, respectively. However, under drought stress only a slight reduction in activity was observed in BARI tomato 16 compared to the stronger reduction observed under salinity.

Overexpressing GLY I transgenic plants can tolerate the salinity stress [43], however, this study used high-yielding tomato varieties that showed reduced GLY I activity in several stress treatments. Also, a previous study showed that overexpression of GLY II in plants leads to enhanced salinity tolerance [44]. Moreover, the GLY I and GLY II enzyme activity depends on the duration of the stress period. Short-term stress can increase enzyme activity by inducing gene transcription, however, long-term stress can decrease enzyme activity [45,46]. Moreover, plants can achieve control value after being exposed to short-term salinity stress and after that being treated with a proper nutrient solution without salt [47]. Additionally, GLY I and GLY II transcripts show an upregulation in leaves, stems, and different parts of the plant after being treated by mannitol for up to 72 hours [48,49]. In our study, we have given salinity and drought stress for 120 hours, which may cause the downregulation of enzyme activity. Therefore, the reduced GLY I, GLY II, and DLDH activities observed in several variety-stress combinations are consistent with a long-term stress response rather than a short-term induction pattern. Prolonged stress can reduce enzyme activity through oxidative damage, substrate limitation, altered protein stability, or reduced metabolic capacity.

Previous studies showed MG as a stress biomarker in plants [42,48] and the downregulation of GLY I and GLY II might indicate reduced MG detoxification-related capacity under prolonged stress. Studies showed that low MG promotes root and shoot growth, whereas high MG concentrations restrict root and shoot growth [50] which also supports root-shoot growth in the salinity and drought conditions compared to the control samples. Moreover, MG can influence plant stress responses by controlling stomatal dynamics, the generation of reactive oxygen species, cytosolic calcium ion levels, the activation of inward rectifying potassium channels, and the expression of numerous stress-responsive genes [9]. Because MG concentration was not directly measured in this study, our results should be interpreted as changes in MG detoxification-related enzyme activity rather than direct evidence of MG accumulation.

DLDH catalyzes D-lactate produced from the MG detoxification pathway. A recent study reported that D-lactate levels increased in plants during abiotic stress conditions [13]. However, there is very little research on the activity of DLDH during abiotic stress conditions. Moreover, as D-lactate is produced from MG through the GLY I and GLY II pathways, its availability depends on the efficiency of these enzymes. During the long-term stress, the reduced activity of GLY I and GLY II might reduce D-lactate production and affect downstream DLDH-linked metabolism. The marked reduction of DLDH activity, especially in BARI tomato 18 under drought and BARI tomato 2 under both stress treatments, suggests that downstream D-lactate metabolism was strongly affected by prolonged salinity and drought stress. In addition, although DLDH is important for MG detoxification, DLDH activity did not show a single consistent varietal pattern across both stresses, suggesting that its response depends on both variety and stress condition. Because MG and D-lactate were not directly quantified, this interpretation remains indirect but provides a useful basis for future mechanistic testing.

### 3.3. *Effect of salinity and drought on H*_*2*_*O*_*2*_ *and proline level*

**Fig 5:**
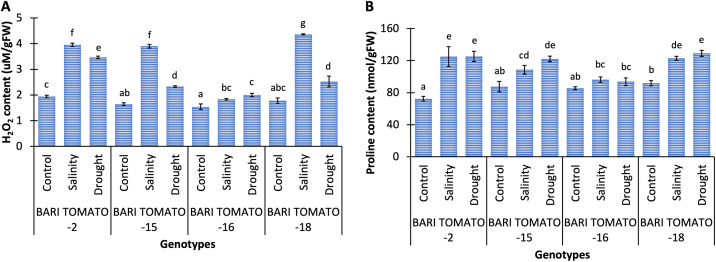
Impact of stress treatment on (A) H_2_O_2_ and (B) proline levels under salinity, drought, and control without any stress treatments on tomato varieties. Different lowercase letters indicate significant differences among variety-treatment combinations based on Tukey’s HSD test (p < 0.05). For H_2_O_2_ and proline, n = 3 per variety-treatment combination. Bars and error bars stand for mean values and standard deviations, respectively.

Abiotic stress can lead to increased production of proline and ROS as signaling molecules [51]. Our study showed that both salinity and drought increased H_2_O_2_ levels in the studied tomato varieties, with salinity generally producing the stronger increase (Fig 5A). Particularly, in BARI tomato 18, H_2_O_2_ level increased by approximately 145% compared to control under salinity condition (Fig 5A). BARI tomatoes 2 and 15 also showed large salinity-induced increases in H_2_O_2_, reaching 3.95 and 3.90 µM/g FW, respectively. Under drought, BARI tomato 2 showed the highest H_2_O_2_ level, with an approximately 79% increase compared with the control. In contrast, BARI tomato 16 exhibited the least H_2_O_2_ accumulation in response to both stress treatments, with increases of nearly 19% and 30% under salinity and drought stresses, respectively. This suggests that there might be variety-dependent oxidative responses as BARI tomato 16 exhibited comparatively less H_2_O_2_ accumulation under both stresses.

Excessive accumulation of ROS, such as H_2_O_2_, can result in oxidative stress, which leads to peroxidation of lipid membranes and oxidation of protein and nucleic acids, consequently resulting in chlorophyll degradation [37,50,51]. Besides the oxidative damage, previous studies showed that ROS are important in plants as signal transduction molecules, mediating responses to pathogen infection, environmental stresses, programmed cell death, and various developmental stimuli [52,53]. Moreover, a recent study showed that increased level of H_2_O_2_ manifested with cell death and decreased biomass [39]. Reduced DLDH activity might be associated with higher H_2_O_2_ accumulation under stress; however, this relationship should be interpreted cautiously because MG, D-lactate, and pyruvate were not directly quantified in this study. DLDH plays a role in MG detoxification by converting D-lactate to pyruvate, which is an important metabolic intermediate involved in antioxidant mechanisms. Pyruvate has been reported to scavenge H_2_O_2_ and protect cells from oxidative injury. Therefore, decreased DLDH activity could cause decreased pyruvate levels, leading to ROS accumulation and enhanced oxidative stress [13]. The decreased DLDH activity with salinity stress noted in this study corresponds with increased H_2_O_2_ production in several varieties. These findings may suggest a relationship between DLDH activity and redox homeostasis during abiotic stress.

This study showed that during salinity and drought treatment, there is a significant increase in proline levels compared to the untreated control samples with a higher accumulation in BARI tomato 2 in both stress conditions and in BARI tomato 18 under drought (Fig 5B). Proline content in BARI tomato 2 increased from 72.32 nmol/g in control to 124.92 nmol/g with salinity and 125.15 nmol/g with drought stress, representing about a 73% increase in proline content with both salinity and drought stress treatments. BARI tomato 18 also had high proline accumulation with stress treatments with proline levels reaching 122.84 nmol/g with salinity and 129.07 nmol/g with drought stress. Two-way ANOVA showed significant effects of tomato variety, stress treatment, and their interaction on proline accumulation (Fig 5B).

During salinity condition, proline can be accumulated due to the enhanced synthesis within the tissue, reduced oxidation, or impaired incorporation of proline into proteins [54]. However, in salinity stress, proline gets accumulated by the simultaneous activation of proline biosynthesis in the cytosol and the suppression of its breakdown in the mitochondria [55]. Moreover, during salinity stress higher Na^+^ concentration can create osmotic stress by reducing water availability and disrupting cellular ion balance [56]. Proline accumulation acts as a defensive mechanism against the osmotic pressure in plant cells [39]. However, higher proline accumulation did not fully prevent stress-related reductions in RWC, chlorophyll content, or enzyme activities in all varieties [57]. Therefore, proline accumulation should be interpreted as an osmotic stress-response marker rather than direct evidence of overall stress tolerance.

Future research should focus on testing these findings under field conditions to determine how prolonged salinity and drought stress affect tomato growth, yield, and fruit quality. Since there is limited study on DLDH activity and its activity decreased under stress, further studies should investigate its gene expression and protein regulation to better understand its role in MG detoxification and oxidative stress reduction [58,59]. Moreover, approaches that maintain or regulate DLDH activity might be explored as potential strategies for improving stress-response capacity, but this requires future research. Further research on the application of osmoprotectants, such as proline or antioxidants, is also needed to determine whether they can help maintain chlorophyll levels and reduce oxidative damage under salinity and drought stress. Also, scientific analysis through transcriptomics combined with metabolomics and proteomics has the potential to deepen the understanding of salinity and drought effects on stress-response pathways to support sustainable agriculture in challenging environments.

These findings indicate that the tested tomato varieties did not respond to salinity and drought through a single uniform pattern. BARI tomato 2 maintained relatively higher shoot growth and showed strong proline accumulation under stress, while BARI tomato 16 showed higher chlorophyll retention and RWC along with lower H_2_O_2_ accumulation. These contrasting profiles provide useful physiological and biochemical markers for future stress-response screening. The integration of GLY I, GLY II, and DLDH activities with growth, water-status, chlorophyll, H_2_O_2_, and proline measurements provides a more complete seedling-stage profile of how high-yield BARI tomato varieties respond to salinity and drought. In particular, the inclusion of DLDH activity adds an underexplored downstream component of MG detoxification-related metabolism to tomato stress profiling. Future studies on quantification of MG and D-lactate directly and evaluation of field performance, yield, and fruit quality are required to determine whether these seedling-stage physiological and biochemical profiles translate into agronomic stress tolerance.

## 4. Conclusion

The effect of salinity and drought stress was assessed on four high-yielding varieties on the basis of morphological parameters like root and shoot length, RWC, chlorophyll contents as well as activity of methylglyoxal detoxifying enzymes and stress markers. The results indicate that the four varieties showed distinct physiological and biochemical responses to salinity and drought rather than a single uniform tolerance pattern. Moreover, root length, shoot length, relative water content, and total chlorophyll were decreased during the abiotic stress conditions while proline and hydrogen peroxide accumulation increased. Enzyme activities also decreased in several variety-treatment combinations after prolonged stress exposure. Interestingly, the substantial reduction of DLDH activity under long-term stress indicated that DLDH is a relevant but underexplored component of MG detoxification-related metabolism under prolonged salinity and drought stress. Seedling-stage growth, water status, chlorophyll, GLY I and GLY II, DLDH, H_2_O_2_ and proline response profiles of four high-yielding BARI tomato varieties were comparatively presented in this study. BARI tomato 2 demonstrated relatively better growth and proline responses, whereas BARI tomato 16 had better chlorophyll retention, RWC and lower H_2_O_2_ accumulation. These differential response profiles are expected to be useful for future stress-response screening and field validations.

## Supporting information

S1 Table Raw data used for figure generation and statistical analyses. (XLSX)

S2 Table Statistical results used for figure annotations. (XLSX)

S1 Checklist Inclusivity in global research questionnaire. (PDF)
